# Continuous auricular acupuncture and daytime intermittent electroacupuncture as a complementary therapeutic improve glycemic variability and stability under Continuous Glycemic Monitoring System in hospitalized diabetic patients

**DOI:** 10.3389/fendo.2025.1716837

**Published:** 2026-01-09

**Authors:** Jianlan Jin, Song Wen, Haiyan Liu, Lijiao Chen, Yishu Ren, Min Gong, Xinlu Yuan, Jiyu Li, Ligang Zhou

**Affiliations:** 1 Department of Endocrinology, Shanghai Pudong Hospital, Fudan University, Pudong Medical Center, Shanghai, China; 2 Department of Nursing, Shanghai Pudong Hospital, Fudan University, Pudong Medical Center, Shanghai, China; 3 Fudan Zhangjiang Institute, Fudan University, Shanghai, China; 4 Department of Surgery, Shanghai Pudong Hospital, Fudan University, Pudong Medical Center, Shanghai, China

**Keywords:** auricular acupuncture, CGMS, diabetes mellitus, electroacupuncture, glycemic variability, personalized treatment

## Abstract

**Objective:**

This study aimed to evaluate the effects of continuous (24h) Auricular acupuncture (AAC) alone and combined with intermittent (20–30 mins daytime, three times per week) Electroacupuncture (EAC) on blood glucose variability (GV) and stability via (Con) CGMS. This approach goes beyond mean glucose levels and could serve as a key determinant of metabolic instability and complication risk in diabetes. The study focused on hospitalized patients with Diabetes Mellitus (DM) and explored their distinct roles across different diurnal segments.

**Methods:**

This retrospective study, conducted at the Department of Endocrinology from 2023 to 2025, analyzed Continuous Glucose Monitoring System (CGMS) data from categorized patient groups (CGMS control, AAC, and AAC+EAC, n=984). Statistical analyses focused on overall mean blood glucose (MBG) and segment-specific (24h, 0-4h, 4-8h, 8-20h, 20-24h) ambulatory glucose profile (AGP) percentiles.

**Results:**

No significant difference in 24-hour MBG was observed among groups (P = 0.9297). Both AAC and AAC+EAC significantly reduced higher glucose percentiles (P75, P90, P95) across 24 hours and during daytime (8-20h) compared to controls. AAC alone was shown to have lower MBG in mid-to-high glucose ranges (P25-P95) during non-EAC periods (0-4h, 4-8h, 20-24h). Both AAC and AAC+EAC groups consistently exhibited significantly higher MBG at lower percentiles (P5, P10) than controls during all segments (0-4h, 4-8h, 20-24h). Notably, during daytime (8-20h), AAC+EAC showed significantly higher P5, P10, and P25 values compared to controls and AAC alone.

**Conclusion:**

Continuous AAC and daytime intermittent EAC effectively reduce glycemic variability in hospitalized patients. AAC provides sustained, broad-spectrum control over glycemic fluctuations in higher percentiles. Daytime EAC uniquely helps prevent lower glucose excursions during its use, indicating a protective role against daytime hypoglycemia. These findings support personalized, complementary interventions for optimized diabetes management.

## 1 Introduction

Diabetes mellitus (DM) remains one of the world’s most significant health challenges, increasing the burden on individuals, healthcare systems, and economies worldwide. Characterized by chronic high blood sugar, it leads to a variety of disabling microvascular complications (1). The World Health Organization (WHO) forecasts its prevalence will continue to increase, emphasizing the urgent need for comprehensive and innovative management strategies to reduce its devastating effects.

The foundation of diabetes management lies in achieving and maintaining optimal glycemic control. This involves not only lowering average blood glucose levels but also minimizing glycemic variability (GV) and increasing time in target glucose range (TIR), which is the percentage of time each day that a person’s glucose level, as measured by continuous glucose monitoring (CGM), remains between 3.9 and 10.0 mmol/L (70–180 mg/dL) (2). High GV, characterized by large fluctuations between hyperglycemia and hypoglycemia, has become an independent risk factor for both acute complications and chronic microvascular issues (3, 4). Therefore, a comprehensive approach to glycemic control includes monitoring the patterns of blood glucose fluctuations throughout the day and night. The American Diabetes Association (ADA) increasingly emphasizes patient-centered care and the use of technologies that empower both patients and clinicians to achieve the best outcomes while reducing risks, especially hypoglycemia. These guidelines form the foundation for evidence-based practice worldwide, ensuring that diabetes care advances with scientific knowledge and clinical experience (5).

The advent and widespread use of continuous glucose monitoring systems (CGMS) have fundamentally changed diabetes management by providing unprecedented insights into glycemic patterns (6, 7). Unlike traditional self-monitoring of blood glucose (SMBG) via fingerstick, CGMS offers continuous, real-time or near real-time glucose readings, trends, highs, lows, and duration within, above, and below target ranges. This extensive data empowers both patients and healthcare providers to detect subtle glycemic patterns that might otherwise go unnoticed, enabling more precise and proactive therapeutic adjustments (8, 9). CGMS guidelines, issued by various professional organizations, recommend its use in patients on intensive insulin therapy, those experiencing problematic hypoglycemia (recurrent, nocturnal, or hypoglycemia unawareness), and individuals with high glycemic variability, among others (10). The data obtained from CGMS, including AGP reports, are invaluable for personalizing treatment plans, optimizing insulin doses, and tailoring lifestyle recommendations, ultimately improving TIR and reducing the risk of both hypo- and hyperglycemia (11).

There is increasing global interest in combining traditional Chinese Medicine (TCM) with modern medical approaches, recognizing their potential for synergistic effects and for offering more holistic, patient-centered care, especially when conventional treatments have limitations or side effects. Auricular acupuncture (AAC), a unique branch of acupuncture, is a micro-acupuncture system based on the idea that the auricle (external ear) is a microsystem reflecting the entire body (12, 13). Proposed mechanisms of AAC in DM include modulation of the autonomic nervous system, regulation of endocrine hormones, improvement of microcirculation, reduction of inflammation, and better glucose utilization (14–17). Its benefits include being non-invasive, relatively simple to apply, and suitable for continuous stimulation (using semi-permanent auricular devices such as 0.2 mm press needles, vaccaria seeds, or magnetic pellets that stay in place for 3–7 days), providing ongoing acupoint pressure with every jaw movement, head turn, or shower. This 24-hour stimulus helps maintain vagal tone and enhances patient compliance compared to single-session body acupuncture (18–20), which may lead to more sustained therapeutic effects and better patient adherence.

Another promising therapeutic approach is Electroacupuncture (EAC). This method involves applying mild electrical currents at low frequencies to specific acupoints, muscles, or nerve pathways (21, 22). In the context of diabetes, EAC is believed to exert its effects through various mechanisms, such as improving local blood flow, stimulating peripheral nerves to influence insulin secretion or sensitivity, increasing glucose uptake by skeletal muscles, and enhancing pancreatic β-cell function (23–25). EAC generally involves intermittent, active stimulation for set durations, meaning the electrical device runs and applies the therapeutic current to the acupuncture points during designated periods. Despite increasing recognition and emerging evidence for AAC and EAC in DM management (14, 26–28), there remains a significant gap in the literature concerning a comprehensive, time-segmented CGMS analysis that directly compares continuous AAC, intermittent daytime EAC, and their combination against standard care in a real-world inpatient setting. This segmentation is crucial to distinguish glycemic control, enabling specific assessment of the dawn phenomenon and the detection of time-specific therapeutic effects, such as protection against daytime hypoglycemia.

This study aims to fill this knowledge gap. We seek to provide new insights into how continuous AAC and intermittent daytime EAC affect glycemic variability and glycemic stability (for example, how precisely and smoothly a patient’s blood glucose levels remain within a target range), using CGMS data. When comparing the individual and combined effects of these approaches against a control group receiving standard care, this research aims to clarify their distinct mechanisms and time-specific impacts on glycemic variability. The results will strengthen the evidence supporting the integration of TCM approaches as complementary therapies alongside standard medical care, guiding personalized treatment strategies that improve glycemic stability and potentially lower the risk of diabetes-related complications, ultimately enhancing the overall well-being of diabetic patients in a comprehensive inpatient setting.

## 2 Materials and methods

### 2.1 Study design and setting

This retrospective study was carried out at the Department of Endocrinology, Shanghai Pudong Hospital, from January 2023 to December 2025. Its purpose was to assess how TCM-based interventions, specifically AAC and EAC, influence glycemic control in hospitalized diabetic patients, measured through CGMS. All information was obtained from the hospital’s electronic medical records. Patient demographic details reviewed retrospectively are shown in **Table 1**.

**Table 1:** The demographic information of retrospective reviewed patients.

	Control group	AAC group	AAC+EAC group	P value
No.	333	319	332	/
Age (yrs)	64.14 ± 15.39	62.15 ± 15.03	62.10 ± 15.48	0.148
Gender(F/M)	182/151	146/173	168/164	0.075
Durations (yrs)	9.19 ± 8.35	8.57 ± 8.26	8.59 ± 9.01	0.569
ADM Drug Use (OADs/Insulin/Combination)	114/17/198	91/13/215	108/20/204	0.323

### 2.2 Participants

#### 2.2.1 Inclusion criteria

A total of 984 hospitalized adult patients with confirmed diabetes mellitus (per WHO criteria) admitted to the Department of Endocrinology were considered for inclusion. Patients were assigned to either standard inpatient care (control group), the continuous AAC group, or the AAC+EAC group. Importantly, only patients with at least 72 hours of valid CGMS data recorded throughout their hospitalization were included.

#### 2.2.2 Exclusion criteria

Patients were excluded if they had: severe acute complications (e.g., diabetic ketoacidosis, hyperosmolar hyperglycemic state) upon admission; severe systemic infections or malignancies; significant cognitive impairment that prevented cooperation; pregnancy; or incomplete medical records or CGMS data. Patients on type 1 diabetes-specific treatments such as continuous subcutaneous insulin infusion (insulin pump) were also excluded to promote a more uniform patient group regarding diabetes management methods.

#### 2.2.3 Patient categorization

Patients were retrospectively divided into three groups based on the treatment received during hospitalization.

##### 2.2.3.1 CGMS Control group

Received standard inpatient diabetes care without AAC or EAC interventions (n=333).

##### 2.2.3.2 Auricular acupuncture group

Received standard inpatient diabetes care plus continuous AAC (n=319).

##### 2.2.3.3 Auricular acupuncture + electroacupuncture group

Received standard inpatient diabetes care plus continuous AAC and daytime intermittent EAC (n=332).

### 2.3 Interventions

#### 2.3.1 Standard inpatient diabetes care

All participants in the study, across all three groups, received standard inpatient diabetes care following the hospital’s established protocols and national guidelines. This included personalized meal planning tailored to calorie needs and glycemic control, conventional anti-diabetic medications (such as insulin, oral antidiabetics like metformin, sulfonylureas, TZDs, SGLT-2 inhibitors, glucokinase activators, and injectable GLP-1 receptor agonists), regular blood glucose monitoring via fingerstick (in addition to CGMS for study groups), and comprehensive diabetes education provided by nursing and medical staff.

#### 2.3.2 Auricular acupuncture

Patients in the AAC group and AAC+EAC group received continuous auricular acupuncture. Disposable auricular vaccaria seeds (or similar pressure beads) (Dongye Medical Equipment, Co Ltd., Hebei, China) were used to stimulate selected acupoints on the ear. The choice of acupoints was based on TCM principles for diabetes management, typically including, but not limited to, points such as Pancreas (CO11), Gallbladder (CO12), Endocrine (CO18), Sanjiao (Triple Energizer, CO17), and ShenMen (Triangle Fossa, TF4) (14, 29). The ear seeds were carefully affixed to the acupoints using adhesive tape, and patients were instructed to press these points gently for 3–5 minutes, 3–5 times daily. The ear seeds were usually changed every 3–5 days by trained nursing staff or TCM practitioners, who held valid certifications from Shanghai Pudong New District in integrative medicine, to ensure effectiveness and hygiene. The duration of AAC application matched the patient’s inpatient stay, providing continuous stimulation during CGMS monitoring.

#### 2.3.3 Electroacupuncture

Patients in the AAC+EAC group received additional Electroacupuncture (DS-MF2B, Dingshi, CO. Ltd. Nanjing, China) performed by a certified nurse who obtained Shanghai Pudong New District authorized integrative medical certificates. This intervention was administered using specialized transcutaneous electrical stimulators. Electrodes were placed on ZuSanli, a specific body acupoint related to glucose metabolism. Multiple studies, including preclinical and clinical investigations, have demonstrated the utility of ZuSanli (ST36) in treating diabetes and related complications such as diabetic neuropathy or gastroparesis (30–32). The stimulation parameters were standardized as follows:

Frequency: 50 Hz ± 1 Hz.

Waveform: A fixed continuous waveform was utilized throughout the study.

Intensity: Adjusted according to the patient’s individual sensory threshold to maintain a comfortable and tolerable level, usually causing a tingling sensation without pain.

Duration: Each session lasted 20–30 minutes.

Frequency of sessions: Conducted three times weekly on non-consecutive days.

Time of day: EAC sessions were strictly held during daytime hours, matching the active periods and likely post-meal times, and were specifically not conducted at night.

### 2.4 Data collection

#### 2.4.1 Continuous glucose monitoring system

CGMS data served as the main outcome measure. For each participant, a continuous glucose monitoring system (Version 3.0, Flash CGMS, Meiqi, China) was inserted subcutaneously, usually in the abdomen or upper arm, upon admission or when starting study interventions. Sensors were worn for at least 72 consecutive hours to gather enough data for both day and night analysis. The Meiqi CGMS device automatically measured and recorded interstitial glucose every 3 minutes. Patients or nursing staff were instructed to perform fingerstick blood glucose tests for calibration as required by the specific Meiqi CGMS protocol. CGMS data, including raw glucose readings, trends, and alerts, were downloaded after sensor removal using dedicated software. Data points marked as erroneous or out of range due to sensor malfunction were excluded from analysis.

### 2.5 Outcome measures

#### 2.5.1 Primary outcome

The main outcome was blood glucose variability and stability, thoroughly evaluated using CGMS-derived metrics. These included:

##### 2.5.1.1 Overall MBG

The average glucose level over the 24-hour monitoring period.

##### 2.5.1.2 Ambulatory glucose profile percentiles

These provide a detailed overview of glucose distribution across different ranges. The following percentiles were analyzed: P5, P10, P25, P50 (median), P75, P90, and P95. Higher percentiles indicate hyperglycemic excursions, while lower percentiles suggest hypoglycemic risk or lower glucose levels.

##### 2.5.1.3 Detection of BG dynamics through segmented time

Specific time segments to evaluate diurnal and nocturnal patterns and the effect of daytime-only EAC.

- 0–4 hours (early morning/pre-dawn)
- 4–8 hours (early morning/dawn period)
- 8–20 hours (daytime/active hours, including EAC application)
- 20–24 hours (evening/post-dinner period)

### 2.6 Statistical analysis

All statistical analyses were conducted using SPSS Statistics version 26.0 (IBM Corp., Armonk, NY, USA), and big-data interpretation was carried out through Jupyter Notebook. The data visualization was done with GraphPad Prism version 9.0 (LLC, 225 Franklin Street, Boston, MA 02110, USA). For CGMS data analysis: Before comparing groups, the normality of distribution for CGMS-derived percentile data was evaluated using visual inspection of histograms and the Shapiro-Wilk test. Homogeneity of variances across groups was checked with Levene’s test. When comparing MBG and AGP percentiles among the three groups, if data were normally distributed and variances were equal, one-way ANOVA was used, followed by Tukey’s Honestly Significant Difference (HSD) *post-hoc* test for pairwise comparisons. If data were normally distributed but variances were unequal, Welch’s ANOVA was applied, followed by the Games-Howell *post-hoc* test. For non-normally distributed data, the Kruskal-Wallis H test was employed, followed by Dunn’s *post-hoc* test with Bonferroni correction for pairwise comparisons. A two-tailed P-value less than 0.05 (P < 0.05) was deemed statistically significant for all tests.

## 3 Results

### 3.1 The analytic data shows there is no significant difference among the three groups regarding the MBG

**Figure 1:**
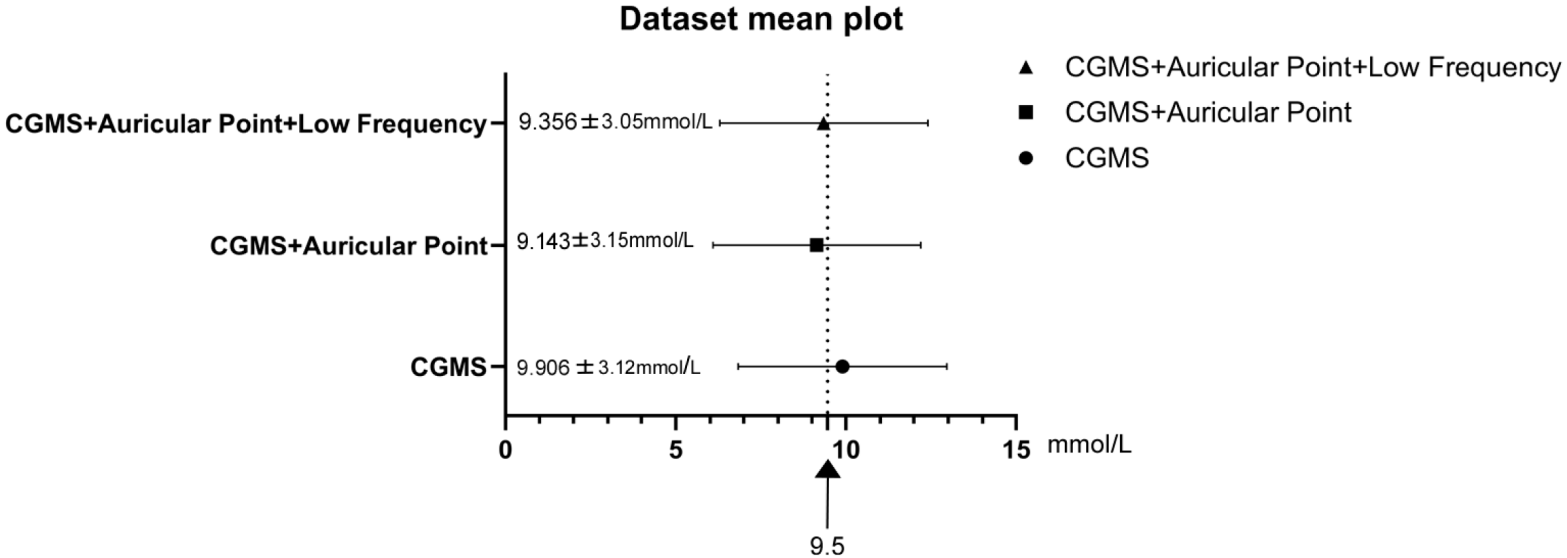
Shows the mean disparity in non-individual 24-hour analyses among three separate groups: the intervention with AAC and/or EAC treatment resulted in lower MBG levels, although this disparity was not statistically significant. CGMS, continuous glucose monitoring system.

Overall analysis of glycemic concentrations showed that the MBG levels were similar across the three intervention groups. Although the MBG improved in the AAC and AAC+EAC groups compared to the CGMS Control group (CGMS+AAC: 9.143 ± 3.15 mmol/L; CGMS+AAC+EAC: 9.356 ± 3.05 mmol/L; CGMS: 9.906 ± 3.12 mmol/L), this difference did not reach statistical significance (p=0.93, ANOVA, HSD *post-hoc* test) (**Figure 1**).

### 3.2 The CGMS data shows that during the diurnal period, patients undergoing AAC and/or EAC therapy exhibited improved glycemic fluctuations and stability

CGMS data clearly showed that patients receiving AAC and/or EAC experienced improved glycemic fluctuations and stability. During the pre-dawn hours (0-4am), the 5% percentile, which indicates lowest blood glucose (BG) levels, was higher in groups receiving AAC+EAC compared to the control group (AAC+EAC vs. Control: 4.55 ± 0.156 mmol/L vs. 4.40 ± 0.169 mmol/L; P<0.001). Furthermore, they prevented further increases in BG levels compared to the control group at various percentiles, except for the 10%. Specifically, at the 25th percentile: Control: 5.87 ± 0.136 mmol/L; AAC: 5.78 ± 0.102 mmol/L; AAC+EAC: 5.87 ± 0.238 mmol/L; with control vs. AAC: P<0.05, and AAC+EAC vs. AAC: P<0.05. At the 50th percentile: Control: 7.19 ± 0.285 mmol/L; AAC: 7.01 ± 0.228 mmol/L; AAC+EAC: 7.18 ± 0.234 mmol/L; with control vs. AAC: P<0.0001, and AAC+EAC vs. AAC: P<0.0001. At the 75th percentile: Control: 9.01 ± 0.400 mmol/L; AAC: 8.71 ± 0.383 mmol/L; AAC+EAC: 9.01 ± 0.356 mmol/L; with control vs. AAC: P<0.0001, and AAC+EAC vs. AAC: P<0.0001. At the 90th percentile: Control: 11.40 ± 0.53 mmol/L; AAC: 10.54 ± 0.668 mmol/L; AAC+EAC: 11.48 ± 0.608 mmol/L; with control vs. AAC: P<0.0001, and AAC+EAC vs. AAC: P<0.0001. Lastly, at the 95th percentile: Control: 12.95 ± 0.654 mmol/L; AAC: 11.87 ± 0.780 mmol/L; AAC+EAC: 12.88 ± 0.706 mmol/L; with control vs. AAC: P<0.0001, and AAC+EAC vs. AAC: P<0.0001. The Kruskal-Wallis H test was used to compare the 5%, 10%, 25%, 50%, and 90% percentiles of BG, while Welch’s ANOVA with Games-Howell *post hoc* test was used for the 75% and 95% percentiles.

During the later dawn phenomenon period (4-8am), the consistently decreasing trend of hyperglycemia measured by AAC and EAC was observed in the 75th to 95th percentiles. At the 75th percentile: Control had 11.00 ± 1.536 mmol/L; AAC had 10.64 ± 1.524 mmol/L; AAC+EAC had 10.93 ± 1.497 mmol/L, with control vs AAC: P<0.0001, and AAC+EAC vs AAC: P<0.0001. At the 90th percentile: Control had 13.49 ± 1.673 mmol/L; AAC had 12.76 ± 1.767 mmol/L; AAC+EAC had 13.41 ± 1.602 mmol/L, with control vs AAC: P<0.0001, and AAC+EAC vs AAC: P<0.0001. At the 95th percentile: Control had 15.08 ± 1.750 mmol/L; AAC had 14.21 ± 1.799 mmol/L; AAC+EAC had 14.98 ± 1.776 mmol/L, with control vs AAC: P<0.0001, and AAC+EAC vs AAC: P<0.0001. The Kruskal-Wallis H test was used to compare the 10%, 25%, and 50% percentiles of blood glucose, while Welch’s ANOVA and the Games-Howell *post-hoc* test were used for the 5%, 75%, 90%, and 95% percentiles.

During the daytime from 8am to 8pm and after dinner, the AAC and EAC groups maintained a trend toward improved hyperglycemia. From 8am to 8pm, the 75th percentile values were: control 11.00 ± 1.536 mmol/L; AAC 10.64 ± 1.524 mmol/L; AAC+EAC 10.93 ± 1.497 mmol/L, P<0.0001. The 90th percentile values were: control 13.49 ± 1.673 mmol/L; AAC 12.76 ± 1.767 mmol/L; AAC+EAC 13.41 ± 1.602 mmol/L, P<0.0001. The 95th percentile values were: control 15.08 ± 1.750 mmol/L; AAC 14.21 ± 1.799 mmol/L; AAC+EAC 14.98 ± 1.776 mmol/L, P<0.0001. The Kruskal-Wallis H test was used to compare the 10%, 25%, 50%, 90%, and 95% percentiles of blood glucose, while Welch’s ANOVA with the Games-Howell *post-hoc* test was used for the 5% percentile.

**Figure 2:**
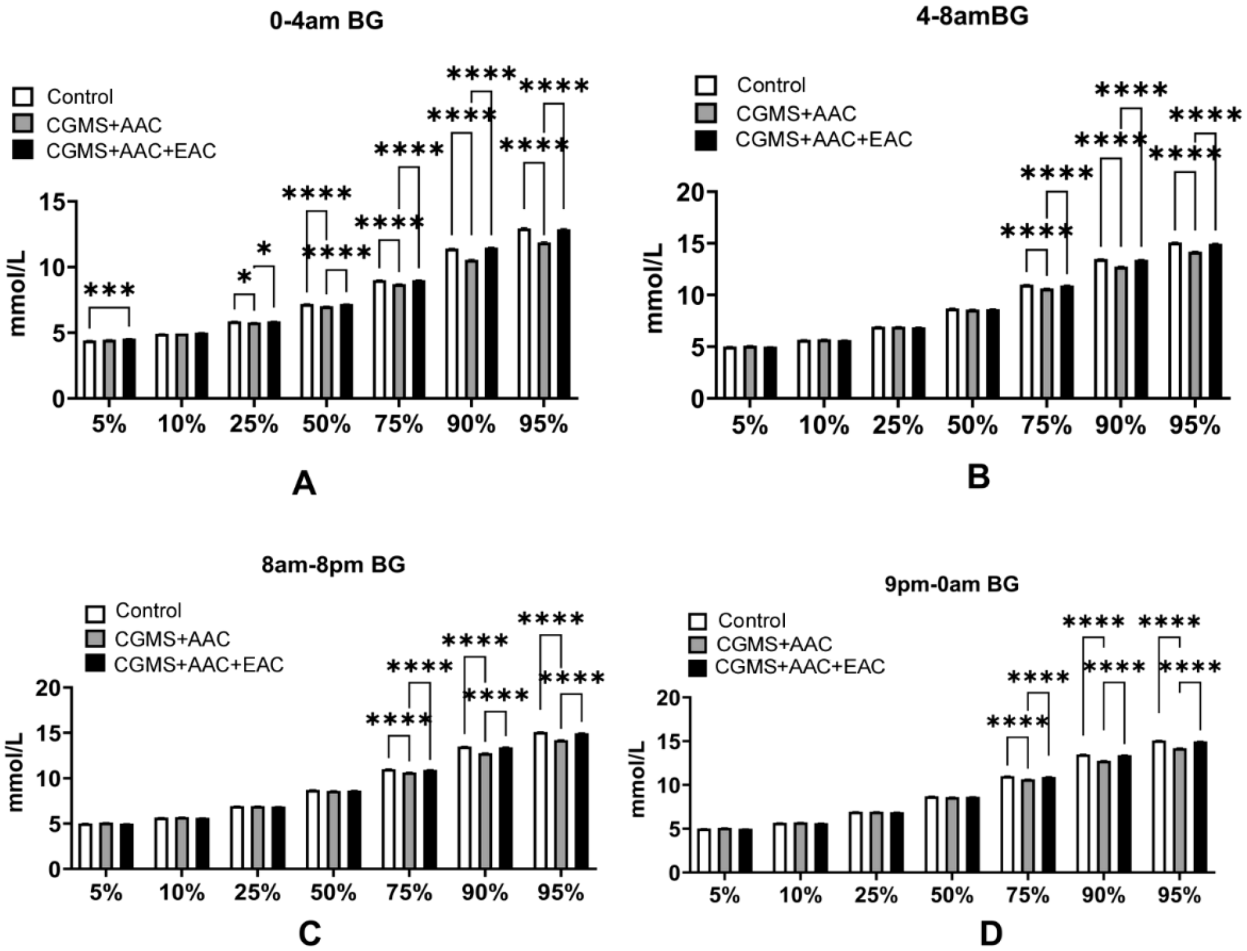
The segmented glucose variation period analysis shows that AAC can prevent hyperglycemic trends throughout the day, while AAC+EAC can prevent hypoglycemic trends during the pre-dawn period. *P<0.05; ***P<0.001; ****P<0.0001; BG, blood glucose; AAC, auricular acupuncture; EAC, electroacupuncture; CGMS, Continuous glycemic monitoring system. **(A)** (0-4am) **(B)** (4-8am) **(C)** (8am-8pm) **(D)** (9pm-0am).

From 9pm to 0am: 75%: Control: 11.00 ± 1.536 mmol/L; AAC: 10.64 ± 1.524 mmol/L; AAC+EAC: 10.93 ± 1.497 mmol/L, P<0.0001; 90%: Control: 13.49 ± 1.673 mmol/L; AAC: 12.76 ± 1.767 mmol/L; AAC+EAC: 13.41 ± 1.602 mmol/L, P<0.0001; 95%: Control: 15.08 ± 1.750 mmol/L; AAC: 14.21 ± 1.799 mmol/L; AAC+EAC: 14.98 ± 1.776 mmol/L, P<0.0001). The Kruskal-Wallis H test was used to compare the 5%, 50%, 75%, 90%, and 95% BG percentiles, while the 10% and 25% BG percentiles were analyzed with Welch’s ANOVA and the Games-Howell *post-hoc* test (**Figure 2**).

### 3.3 The overall analysis of glucose profiles across the three groups revealed improved glycemic variability in the AAC and/or EAC treatment groups

**Figure 3:**
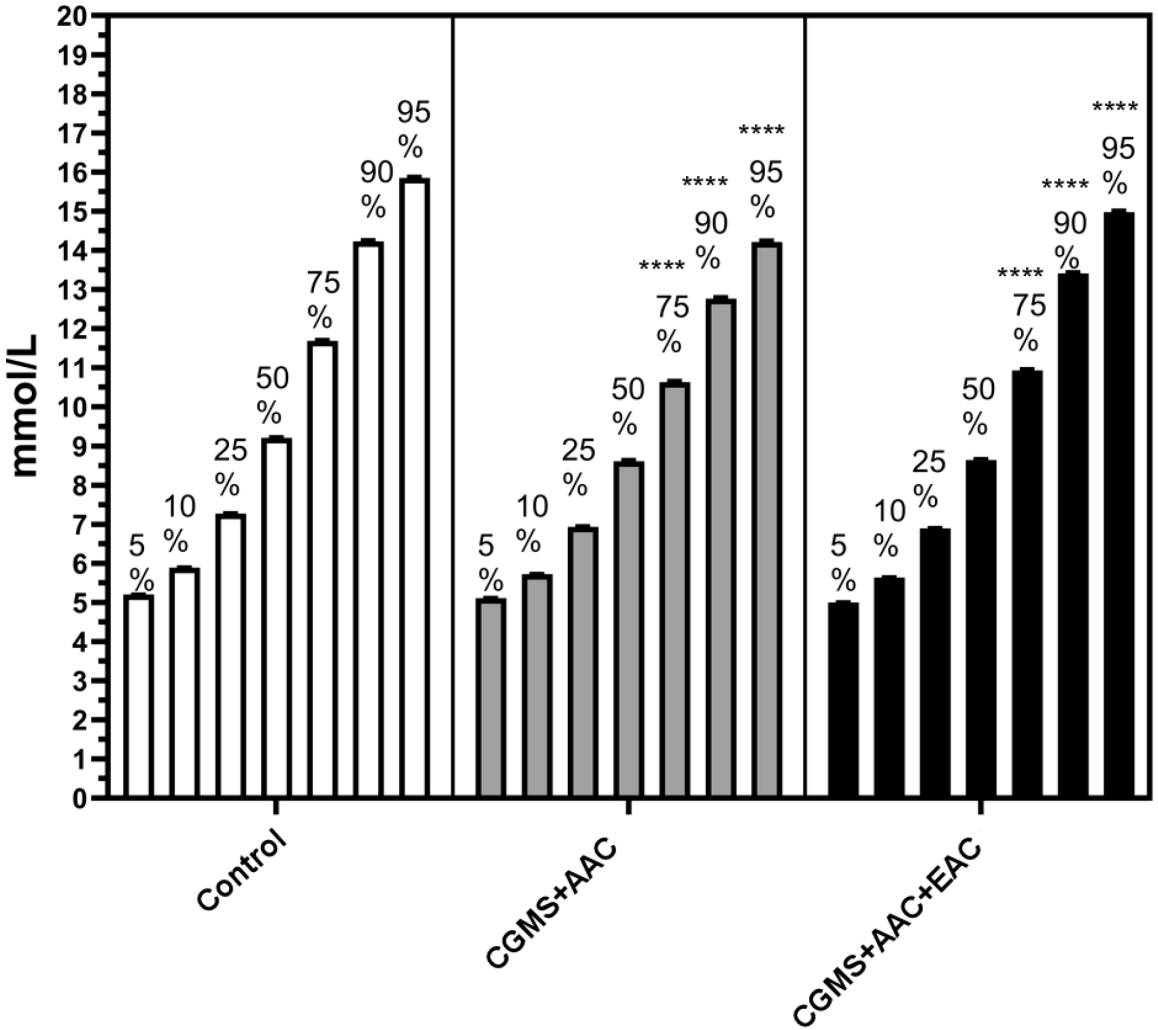
The improved high percentile glycemic variation (75-95%) within 3 days was observed among the AAC group and adjunctive EAC treatment group, analyzed via nest methods. ****p<0.0001: compared with control group. AAC, auricular acupuncture; EAC, electroacupuncture.

The overall nested analysis of the glucose profile across the three intervention groups clearly showed improved glycemic variability in the AAC and/or EAC treatment groups. This improvement is particularly evident in the significantly reduced 75% to 95% percentile of glycemic variation. In contrast, there were no significant differences among the three groups in the modest, median, or low percentiles of glycemic variation (**Figure 3**). The Kruskal-Wallis H test was used to compare the 5%, 10%, 25%, 50%, and 90% percentiles of blood glucose (BG). Conversely, the 75% and 95% percentiles were analyzed using Welch’s ANOVA and the Games-Howell *post-hoc* test.

### 3.4 The overall analysis of time in range among the three groups showed improved TIR in the AAC and/or EAC treatment groups

**Figure 4:**
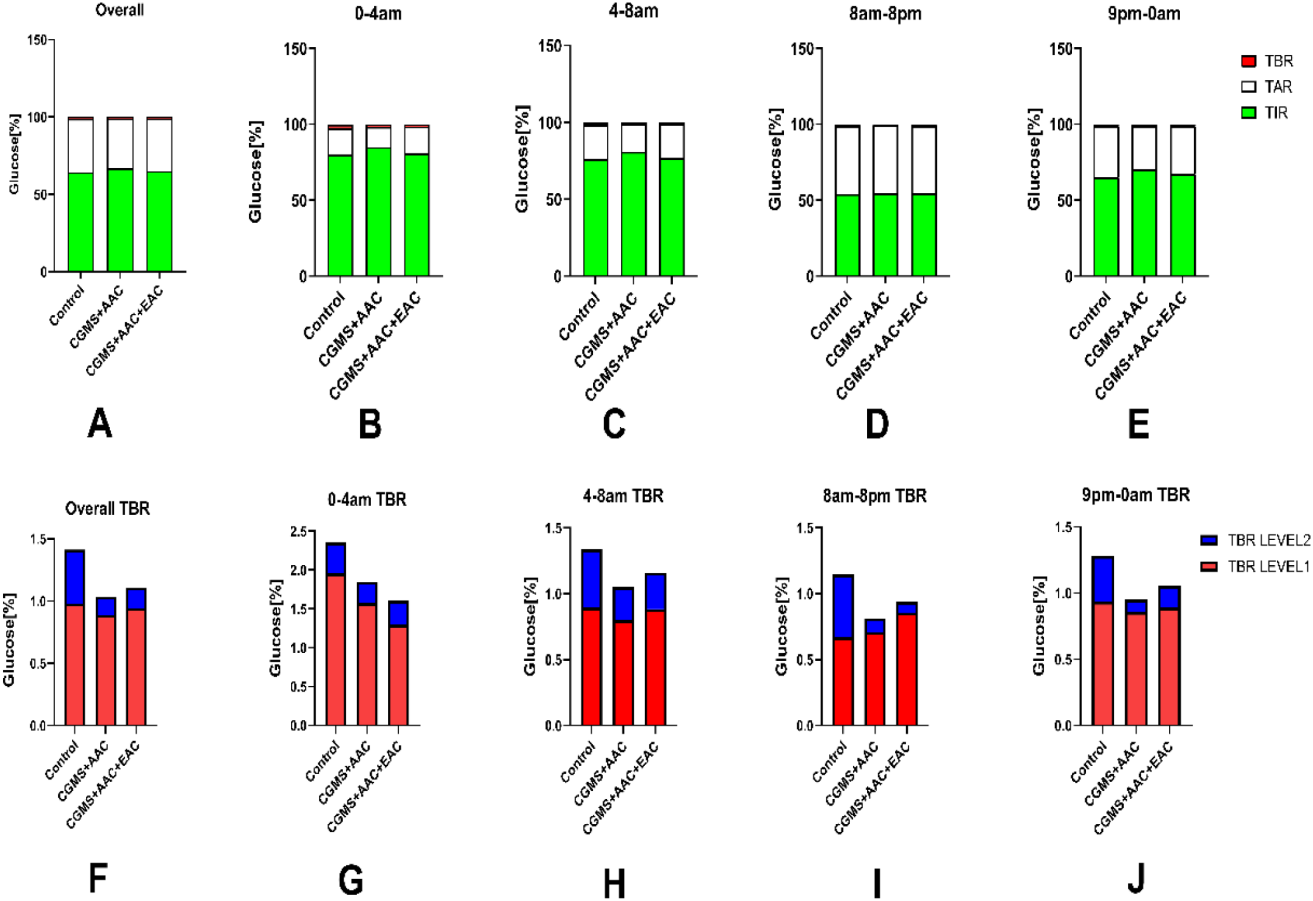
The AAC and EAC could maintain glycemic stability by lowering the mean BG but decreasing the incidence of hypoglycemic events, as evidenced by the improvement in TIR **(A–E)**, and reductions in TAR, TBR, TBR level 1, and TBR level 2 **(F–J)**. Note: TIR: percentage of time in euglycemia (BG: 3.9-10.0 mmol/L); TAR: percentage of time in hyperglycemia (BG >10.0 mmol/L); TBR: percentage of time in hypoglycemia (BG <3.9 mmol/L); TBR Level 1: percentage of time in Level 1 hypoglycemia (3.0 mmol/L < BG < 3.9 mmol/L); TBR Level 2: percentage of time in Level 2 hypoglycemia (BG <3.0 mmol/L). CGMS, continuous glycemic monitoring system; AAC, auricular acupuncture; EAC, electroacupuncture.

The overall analysis of TIR among the three groups showed a significant improvement in TIR for the AAC and/or EAC treatment groups. As shown in **Figure 4**, the AAC group achieved a significantly higher overall TIR percentage (Control: 64.18%; AAC: 66.72%; AAC+EAC: 65.08%), along with reduced TAR (Control: 34.41%; AAC: 32.25%; AAC+EAC: 33.81%) (**Figure 4A**), and lower TBR (Control: 1.41%; AAC: 1.03%; AAC+EAC: 1.11%). TBR levels 1 and 2 were also decreased (Level 1: Control: 0.98%; AAC: 0.88%; AAC+EAC: 0.94%; Level 2: Control: 0.43%; AAC: 0.15%; AAC+EAC: 0.17%) (**Figure 4F**). Additionally, during the 0–4 am period, TIR was higher in the AAC and AAC+EAC groups (Control: 79.82%; AAC: 84.83%; AAC+EAC: 80.75%), with lower TAR (Control: 17.52%; AAC: 13.32%; AAC+EAC: 17.64%), and decreased TBR and TBR levels 1 and 2 (Control: 2.35%; AAC: 1.85%; AAC+EAC: 1.61%; TBR Level 1: Control: 1.96%; AAC: 1.57%; AAC+EAC: 1.30%; TBR level 2: Control: 0.39%; AAC: 0.27%; AAC+EAC: 0.31%) (**Figures 4B, G**). During the 4–8 am period, similar trends were observed: higher TIR (Control: 76.14%; AAC: 80.79%; AAC+EAC: 76.93%), with lower TAR (Control: 22.23%; AAC: 18.16%; AAC+EAC: 21.91%), and reduced TBR and TBR levels 1 and 2 (Control: 1.34%; AAC: 1.05%; AAC+EAC: 1.16%; TBR Level 1: Control: 0.90%; AAC: 0.80%; AAC+EAC: 0.88%; TBR level 2: Control: 0.44%; AAC: 0.25%; AAC+EAC: 0.28%) (**Figures 4C, H**). In the 9 pm to 0 am period, TIR remained higher in the treatment groups, with the following percentages: Control: 65.27%; AAC: 70.37%; AAC+EAC: 67.52%. TAR was lower in these groups as well (Control: 33.45%; AAC: 28.68%; AAC+EAC: 31.42%). TBR and TBR levels 1 and 2 were also reduced (Control: 1.28%; AAC: 0.95%; AAC+EAC: 1.06%; TBR Level 1: Control: 0.94%; AAC: 0.86%; AAC+EAC: 0.89%; TBR Level 2: Control: 0.34%; AAC: 0.09%; AAC+EAC: 0.16%) (**Figures 4E, J**). Similar trends were observed overall. However, during daytime hours (8 am-8 pm), no significant advantage over TAR was found with AAC, and TBR level 1 showed no difference, although other indices improved (TIR: Control: 54.10%; AAC: 54.35%; AAC+EAC: 54.66%; TAR: Control: 44.75%; AAC: 44.84%; AAC+EAC: 44.41%; TBR: Control: 1.14%; AAC: 0.81%; AAC+EAC: 0.94%; TBR Level 1: Control: 0.67%; AAC: 0.71%; AAC+EAC: 0.86%; TBR level 2: Control: 0.47%; AAC: 0.10%; AAC+EAC: 0.08%) (**Figures 4D, I**). When analyzing mean and median values, glucose levels did not show statistically significant differences across time blocks (p>0.05). The Kruskal-Wallis H test was used to compare TIR, TBR, and TAR because their distributions were non-normal and variances were unequal. The comparison of mean and median BG also used the Kruskal-Wallis H test due to unequal variances.

### 3.5 The ambulatory glucose profile analysis shows improved glucose variability in AAC and/or EAC treatment groups

**Figure 5:**
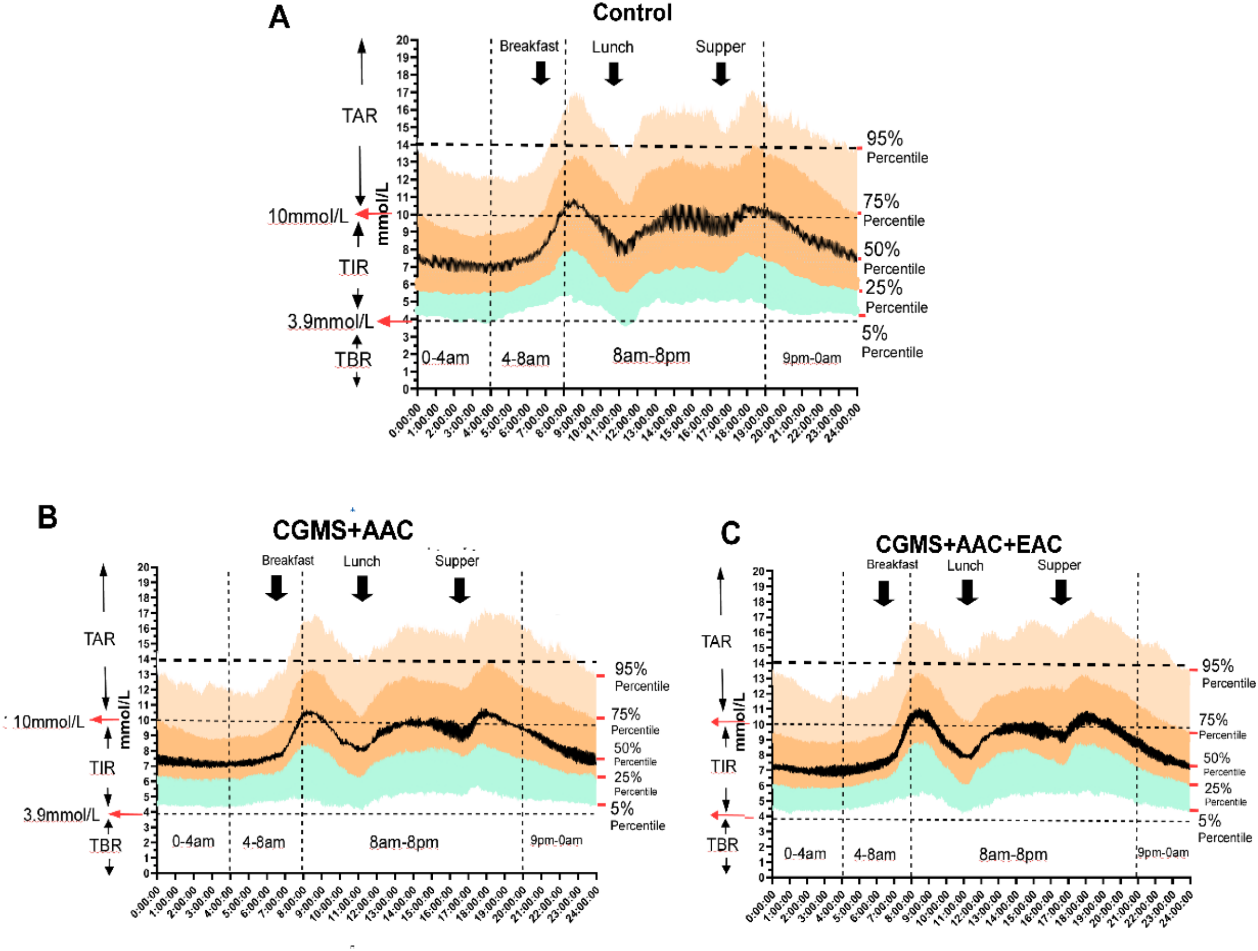
The AGP figure (proportions and probability distributed in 24 hours of BG) shows the improvement of glycemic variation in treatment groups of AAC and EAC, represented by attenuated fluctuations in nocturnal glycemia (including glucose excursions within the time segments of 9 pm-0 am, 0 am-4 am, and 4 am-8 am), reduced extreme postprandial hyperglycemia (the glucose excursions after each of the 3 black arrows indicating postprandial glucose), as well as the pattern of changes in glycemia. The ranges of TIR, TBR, and TAR are shown on the left axis, while the horizontal dotted lines indicate the hypoglycemic boundary (3.9 mmol/L), the Level 1 hyperglycemic boundary (10 mmol/L), and the Level 2 hyperglycemic boundary (13.9 mmol/L). The vertical dotted lines mark the time intervals of 12:00am-4:00am, 4:00am-8:00am, 8:00pm-8:00pm, and 8:00am.-12:00am. On the right side of each subfigure, the glucose percentiles are listed as 95%, 75%, 50%, 25%, and 5%, respectively. The three black arrows indicate the times of food ingestion. **(A–C)** represented the Control Group, AAC and EAC groups, respectively.

A comprehensive AGP analysis consistently showed improved glucose variability in the AAC and/or EAC treatment groups throughout the 24-hour cycle. Specifically, compared to the control treatment group (**Figure 5A**), the AAC (**Figure 5B**) and AAC+EAC (**Figure 5C**) groups consistently exhibited significantly better hyperglycemia at the 75% and 95% percentiles in the CGMS+AAC and CGMS+AAC+EAC groups, respectively, compared to the control. They also showed improved hypoglycemic profiles at the 5% percentile (p < 0.0001), indicating effective mitigation of hyperglycemic excursions without increasing hypoglycemia risk. Collectively, these AGP findings strongly suggest that these interventions lead to more compressed and stable glucose profiles throughout the day, effectively reducing overall glycemic fluctuations and optimizing patient glycemic stability.

## 4 Discussion

This study examined the effects of AAC and AAC+EAC on glycemic control and variability, as measured by CGMS data, compared to a control group. The results offer insights into how these interventions affect different aspects of glucose profiles over various time periods.

Multiple clinical studies have examined the efficiency and reliability of CGMS in assessing treatment safety and BG fluctuations in Type 1 and Type 2 DM and other BG management situations (11, 33–36). The overall analysis of glucose concentrations indicated that, while MBG levels were numerically lower in the AAC and AAC+EAC groups compared to the CGMS Control group (CGMS+AAC: 9.143 mmol/L; CGMS+AAC+EAC: 9.356 mmol/L; CGMS: 9.906 mmol/L), this difference did not reach statistical significance (p=0.93). The possible explanations accounted for this effect may be the antidiabetic treatment, including pharmacological interventions, lifestyle interventions, insulin secretion, and insufficient state, insulin sensitivity, liver glucose output, as well as the basic demographic homogeneity of the included patients in the three groups, respectively. This suggests that although the average glucose level was generally similar, the primary impact of these TCM interventions may be more pronounced on the pattern and variability of blood glucose rather than solely on the overall mean value (33, 37–39).

The CGMS data clearly showed that patients undergoing AAC and/or EAC experienced improved glycemic fluctuations and stability, especially during the daytime hours. This was strongly supported by the percentile analysis. During the pre-dawn period (0-4am), AAC+EAC significantly prevented a rise toward hypoglycemia at the 5th percentile (4.55 ± 0.156 mmol/L vs. 4.40 ± 0.169 mmol/L for AAC+EAC; p<0.001), while also preventing further increases in glucose at other percentiles (25%, 50%, 75%, 90%, 95%) compared to the control group, except for the 10th percentile. Similarly, during the late dawn phenomenon period and throughout the daytime (8am-8pm) and post-dinner hours (9pm-0am), a consistent and statistically significant decrease in hyperglycemic trends was observed at the 75th, 90th, and 95th percentiles in the AAC and AAC+EAC groups (all p<0.0001). Also, as the later analyses showed, the TBR of the segmented 24-hour analysis period and the AGP profile improved the proportion of hypoglycemia in AAC and AAC+EAC (**Figures 4**, **5**). These findings highlight how these interventions can reduce glucose fluctuations, especially by lowering hyperglycemic peaks, which helps improve overall glycemic stability.

The overall analysis of the glucose profile across the three groups strongly indicated improved glycemic variability in the AAC and/or EAC treatment groups. This improvement was mainly seen in the significantly reduced 75th-95th percentile range, with no significant differences in the modest, median, or lower percentiles. This specific enhancement in regulating the upper end of glucose fluctuations suggests a targeted effect on hyperglycemia, which is a key aspect of overall glycemic variability and long-term diabetes management.

Furthermore, the overall analysis of TIR showed a significant improvement for the AAC and/or EAC treatment groups. The AAC group achieved a notably higher overall TIR percentage (66.72%) compared to the Control group (64.18%), and AAC+EAC also demonstrated an improved TIR (65.08%), along with reduced TAR and TBR, including Level 1 and Level 2 hypoglycemia (**Figure 4**). Notably, the AAC group showed a particularly significant reduction in TBR Level 2 (0.15% vs. Control: 0.43%), indicating a lower occurrence of severe hypoglycemia. Similar positive trends in TIR, TAR, and TBR were observed across specific time segments. However, it is important to note that in the 8 am-8 pm daytime period, AAC did not demonstrate a clear advantage over TAR, nor for TBR Level 1, although other indices such as TIR still improved. This may be due to the daytime hours from 8am to 8pm, during which the effects of patient activities, pharmaceuticals, and eating behaviors were significant. The glycemia profiles across the groups were similar, and the AAC and EAC did not have the same profound effect on glycemic stability and variability as they did during the night. These results collectively indicate that AAC and EAC can help maintain glycemic stability by improving glucose excursions in both P95% and P75% hyperglycemia, as well as reducing the incidence of hypoglycemic events, including TBR and P5% hypoglycemia. It is important to note that the overall TIR percentages observed in this study (e.g., ~64-67%) reflect glycemic status during a stable alternative assistive treatment process and are based on a relatively short 3-day CGMS monitoring period; therefore, the consistent and long-term therapeutic effects of AAC and EAC require further investigation.

The comprehensive AGP analysis further supported these findings, showing consistently improved glucose variability in the AAC and/or EAC treatment groups throughout the 24-hour cycle. A key strength of this analysis was the use of population-level AGP data, which offers a reliable overview of collective glycemic patterns by averaging and smoothing individual differences across the study group. Specifically, the AAC and AAC+EAC groups consistently demonstrated significantly lower glucose values at higher percentiles (e.g., 75th, 90th, 95th percentiles across all time segments, p < 0.0001), indicating effective reduction in hyperglycemic excursions. At the same time, glucose values at lower percentiles (e.g., 5th, 10th percentiles) did not show significant worsening or remained stable, suggesting they did not raise hypoglycemia risk. These AGP results strongly indicate more compressed and stable glucose profiles throughout the day, helping to reduce overall glycemic fluctuations and improve patient stability. The AGP visualization (**Figure 5**) further illustrates decreased nocturnal glycemia fluctuations and fewer extreme postprandial hyperglycemia episodes, highlighting a positive shift in glycemic patterns.

The observed improvements in glycemic stability and variability warrant exploring potential underlying mechanisms from both clinical and biomedical perspectives. Clinically, AAC regulates BG by modulating stress, sleep patterns, and overall autonomic balance, as these factors are known contributors to glycemic instability (15, 16). EAC has been clinically investigated for its effects on glucose profiles (18) and may influence peripheral glucose uptake or insulin sensitivity. Its additional benefits in chronic metabolic conditions like obesity have also been systematically reviewed (19).

Transcutaneous auricular vagus nerve stimulation (taVNS), a likely mechanism for AAC, directly affects the autonomic nervous system (40–43). The vagus nerve plays a vital role in regulating glucose balance by influencing pancreatic function and hepatic glucose production. Specifically, taVNS has been shown to modulate blood glucose through intestinal melatonin receptors and secretion in animal models (15), and by releasing extrapineal melatonin (16). Melatonin is a hormone known to influence circadian rhythms and glucose metabolism. Studies have demonstrated its ability to correct nerve conduction velocity deficits in diabetic rats (17), indicating a direct impact on diabetic neuropathy, which can impair glucose regulation.

### 4.1 Strengths and limitations

A key strength of this study is the use of high-resolution CGMS data, which offers a detailed 24-hour glucose profile, allowing for in-depth analysis of various glycemic metrics across specific time periods. This supports a nuanced evaluation of treatment effects.

A key limitation of this study is the lack of additional clinical baseline characteristics and follow-up data. This gap hinders the ability to account for potential confounding factors and to investigate links between CGMS metrics and other clinical parameters. As a result, while the findings regarding CGMS outcomes are solid, their broader applicability needs further confirmation. Additionally, the short 3-day duration of CGMS monitoring restricts the evaluation of long-term glycemic patterns and the durability of the observed improvements.

### 4.2 Clinical implications and future directions

The results indicate that AAC, especially when combined with EAC, could be a valuable adjunct therapy for better glycemic control, mainly by improving glycemic variability and increasing TIR. The ability of these interventions to reduce hyperglycemic excursions without significantly raising the risk of hypoglycemia is a significant clinical advantage. Future research should feature longer intervention periods and include a comprehensive range of clinical and biochemical data to verify these findings and clarify the underlying physiological mechanisms.

## Data Availability

The original contributions presented in the study are included in the article/supplementary material. Further inquiries can be directed to the corresponding authors.
